# Extremely divergent COI sequences within an amphipod species complex: A possible role for endosymbionts?

**DOI:** 10.1002/ece3.9448

**Published:** 2022-10-27

**Authors:** Eunji Park, Robert Poulin

**Affiliations:** ^1^ Department of Zoology University of Otago Dunedin New Zealand; ^2^ Department of Botany University of British Columbia Vancouver British Columbia Canada

**Keywords:** cryptic species, intracellular parasites, morphological conservatism, species delimitation, substitution saturation

## Abstract

Some heritable endosymbionts can affect host mtDNA evolution in various ways. Amphipods host diverse endosymbionts, but whether their mtDNA has been influenced by these endosymbionts has yet to be considered. Here, we investigated the role of endosymbionts (microsporidians and *Rickettsia*) in explaining highly divergent COI sequences in *Paracalliope fluviatilis* species complex, the most common freshwater amphipods in New Zealand. We first contrasted phylogeographic patterns using COI, ITS, and 28S sequences. While molecular species delimitation methods based on 28S sequences supported 3–4 potential species (N, C, SA, and SB) among freshwater lineages, COI sequences supported 17–27 putative species reflecting high inter‐population divergence. The deep divergence between NC and S lineages (~20%; 28S) and the substitution saturation on the 3rd codon position of COI detected even within one lineage (SA) indicate a very high level of morphological stasis. Interestingly, individuals infected and uninfected by *Rickettsia* comprised divergent COI lineages in one of four populations tested, suggesting a potential influence of endosymbionts in mtDNA patterns. We propose several plausible explanations for divergent COI lineages, although they would need further testing with multiple lines of evidence. Lastly, due to common morphological stasis and the presence of endosymbionts, phylogeographic patterns of amphipods based on mtDNA should be interpreted with caution.

## Graphical abstract

We contrasted phylogeographic patterns and species delimitation results shown by COI and 28S sequences in an amphipod species complex and explored the potential influence of endosymbionts in mtDNA patterns. Amphipod individuals infected and uninfected by *Rickettsia* comprised divergent COI lineages in one of four populations tested, suggesting a potential impact of endosymbionts, but further investigation is needed since several alternative scenarios exist.

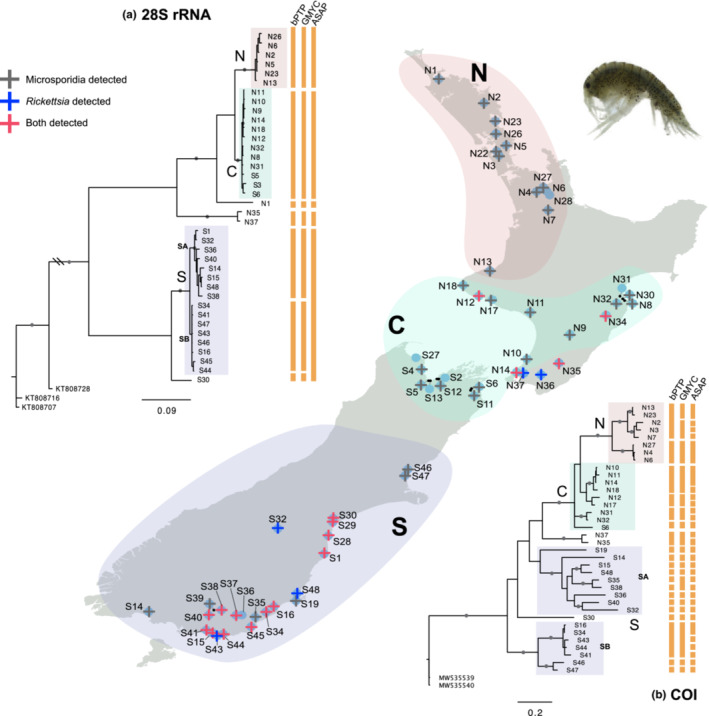

## 1 INTRODUCTION

With more accessible and affordable molecular tools for sequencing, detection of genetically divergent lineages among morphologically similar or indistinguishable organisms has been increasing, often leading to report of cryptic species. (Després, 2019; Gompert et al., 2008; Hinojosa et al., 2019; Toews & Brelsford, 2012). Cryptic species can be defined as “two or more distinct species that are erroneously classified under one species name due to morphological similarities” (Bickford et al., 2007; Struck et al., 2018). Various processes have been suggested as mechanisms to explain the occurrence of cryptic species including recent divergence, parallelism, convergence, and morphological stasis (Struck et al., 2018), with the latter usually invoked for cases with deep genetic divergence (Cerca et al., 2020; Fišer et al., 2018; Lee & Frost, 2002). However, what drives genetic differentiation in the absence of morphological differentiation still remains largely unknown; some potential factors that have been proposed include geographical isolation and changes in behavior or chemical signals (Hogg et al., 2006; Sutherland et al., 2009).

Cryptic species are common in amphipods; some amphipods show high intra‐ and interspecific genetic divergence despite little or no morphological differentiation (Fišer et al., 2018; Havermans et al., 2011; Witt et al., 2006). *Paracalliope fluviatilis*, the most common “morphospecies” in New Zealand freshwaters, is known to comprise several cryptic species based on their deeply divergent cytochrome c oxidase subunit I (COI) sequences, the most commonly used molecular marker for DNA barcoding, in the absence of morphological differentiation (Hogg et al., 2006; Sutherland et al., 2009). While identifying amphipod species using the COI marker, we also observed extremely divergent COI sequences among populations (~24%), among adjacent populations (~22%), and even within a population (~13%) far exceeding the general degree of intraspecific genetic divergence in other taxa (Costa et al., 2009; Hebert et al., 2003; Park et al., 2020; Raupach & Radulovici, 2015). Although geographic isolation among populations due to the limited dispersal abilities of amphipods has been suggested to explain the presence of deeply divergent lineages among populations of *Paracalliope* (Hogg et al., 2006), this cannot explain the presence of divergent lineages in the same location.

There are increasing cases where deeply divergent mtDNA is not supported by nuDNA (Bernardo et al., 2019; Giska et al., 2015; Hinojosa et al., 2019; Kvie et al., 2013; Zhang et al., 2019). This is a special case of mitonuclear discordance, a phenomenon that can be generally defined as “a significant difference in the patterns of differentiation between mitochondrial and nuclear markers” (Toews & Brelsford, 2012). Several explanations have been proposed specifically for deep divergence. For example, deeply divergent mtDNA lineages showing a lack of differentiation in the nuclear genome can sometimes be explained by ancient hybridization events (Brennan et al., 2016; Dupuis & Sperling, 2020; Tóth et al., 2017; Zhang et al., 2019). In cnidarians and copepods, substitution saturation (hence providing little phylogenetic information) has been suggested as a responsible factor for incongruent topologies (Pratlong et al., 2017; Thum & Harrison, 2009). Fundamentally, mtDNA and nuDNA have different natures of inheritance: haploidy and uniparental inheritance for mtDNA in contrast to diploidy and biparental inheritance for nuDNA (Ballard & Whitlock, 2004; Wolff et al., 2014).

Because of maternal inheritance, mtDNA may be influenced by endosymbionts that can be vertically transmitted and manipulate host reproduction (Ballard & Whitlock, 2004; Hurst & Jiggins, 2005; Jiggins, 2003). Some inherited symbionts can spread quickly within a population by increasing the proportion of the transmitting sex (i.e., female) in several different ways (Engelstädter & Hurst, 2009; Werren et al., 2008). Because both mitochondria and endosymbionts are maternally transmitted, they might not be independent but can be associated. As a result, endosymbionts along with their associated mitochondrial haplotype can increase in frequency within a population. This phenomenon, that is, a selective sweep, could happen rapidly and result in reduced mtDNA diversity in an infected population (Hurst & Jiggins, 2005). If multiple strains are present in a population, multiple haplotypes will be maintained, resulting in increased mtDNA diversity within a population (James & Ballard, 2000; Schulenburg et al., 2002). Such patterns arising due to non‐random association between endosymbionts and mtDNA are well known in diverse insect and isopod hosts of the bacteria *Wolbachia* (Bouchon et al., 1998; Werren et al., 2008). In fact, there are at least six bacterial endosymbionts associated with reproductive manipulation; *Arsenophonus*, *Cardinium*, Flavobacteria, *Rickettsia*, *Spiroplasma*, and *Wolbachia* (Cordaux et al., 2011; Duron et al., 2008). Although they are eukaryotes, Microsporidia are also reproductive manipulators capable of feminizing their host (Terry et al., 1998).

Recently, Microsporidia and *Rickettsia*, two known manipulators of host reproduction, were detected in multiple *Paracalliope* populations (Park et al., 2020; Park & Poulin, 2020). Microsporidians are among the most common eukaryotic parasites in amphipods (Bojko & Ovcharenko, 2019). All microsporidians sequenced from *Paracalliope* belonged to the genus *Dictyocoela* (*Dictyocoela* sp. NZ1 ~ 4), the most common microsporidian genus in amphipods in Europe, too (Park et al., 2020). Although feminizing abilities of microsporidians have been investigated (Terry et al., 1998), their possible impact on host mtDNA evolution remains unknown. Moreover, sex ratio distortion and its consequences for mtDNA evolution are known in insect hosts of *Rickettsia* (Hagimori et al., 2006; Schulenburg et al., 2002). Because the presence of *Rickettsia* has only just recently been confirmed in amphipod hosts (Park & Poulin, 2020), their impact on host mtDNA evolution is unknown. All *Rickettsia* sequenced from *Paracalliope* belong to Torix group, which is a sister group to all other groups within the genus *Rickettsia* (Park & Poulin, 2020). They are known to be common in aquatic environments, but little is known about their impact on their hosts. Here, we investigate patterns and causes of extremely divergent COI sequences of *Paracalliope* with special attention to a potential role for endosymbionts. We start by contrasting phylogeographic patterns using COI, 28S ribosomal RNA, and internal transcribed spacer (ITS1‐5.8 S rRNA‐ITS2; ITS) sequences. Also, we delineated species boundaries using several available molecular methods. We examine whether Microsporidia or *Rickettsia* are non‐randomly associated with certain COI haplotypes within a population. Lastly, we discuss how endosymbionts, host life‐history traits, and ancient hybridization events might potentially and synergistically affect mtDNA evolution in amphipods.

## 2 MATERIALS AND METHODS

### 2.1 Host phylogeography

#### 2.1.1 Sample collection and DNA extraction

Amphipod specimens and extracted genomic DNA samples of the *Paracalliope* species complex including their endosymbionts, which were collected from 59 locations throughout New Zealand, were obtained from our previous studies (Park et al., 2020; Park & Poulin, 2020). Specimens were collected with fine‐mesh nets and then preserved in 96% ethanol on site. Amphipod genomic DNA was extracted from several appendages per individual with the Qiagen DNeasy Blood & Tissue kit following the manufacturer's protocol.

#### 2.1.2 PCR

In order to compare phylogenetic patterns between mtDNA and nuDNA, one mitochondrial gene (COI) and two nuclear genes (28S and ITS) were sequenced. ITS sequences were only obtained from a subset of samples. Although 28S sequences were already obtained from 27 locations (one sequence per location) by Park et al. (2020), sequences from 11 further locations were additionally obtained for the present study (one sequence per location), for better geographical coverage. PCR reactions were conducted as described in Park et al. (2020). Since we obtained untargeted rickettsial sequences using universal COI primers, blocking primers were added to reliably amplify host COI sequences (Park & Poulin, 2020). For PCR reactions for the COI region, 11.9 μl of distilled water, 4 μl of reaction buffer, 0.8 μl of each forward and reverse primers, 0.4 μl of blocking primer (DPO_HCO), 0.1 μl of MyTaq (Bioline), and 2 μl of DNA were used. PCR conditions were the following: 95°C for the initial denaturation of 3 min, 5 cycles at 94°C for 60 s, 45°C for 90 s, 72°C for 40 s, 35 cycles at 94°C for 60 s, 48°C for 90 s, 72°C for 40 s, the final extension for 5 min at 72°C. ITS sequences were obtained using ITS_amp_F (5′‐AACCTGCGGAAGGATCATTA‐3′) and ITS_amp_R primers (5′‐ATGCTTAAGTTCAGCGGGTAGTCCC‐3′). For PCR reactions for the ITS region, 12.3 μl of distilled water, 4 μl of reaction buffer, 0.8 μl of each forward and reverse primers, 0.1 μl of MyTaq (Bioline), and 2 μl of DNA were used with the following PCR conditions: 95°C for the initial denaturation of 5 min, 35 cycles at 94°C for 30 s, 55°C for 30 s, 72°C for 80 s, and the final extension for 7 min at 72°C. After PCR reactions, 2 μl of PCR product from each PCR reaction was run on a 1.5% agarose gel.

#### 2.1.3 Sequencing

All PCR products obtained in this study were purified with MEGAquick‐spinTM Total Fragment DNA Purification Kit (iNtRON Biotechnology) according to the manufacturer's instructions. Purified PCR products were sent to Genetic Analysis Services at the University of Otago, New Zealand. Raw nucleotide sequences were carefully examined and corrected by eye, and the existence of nuclear mitochondrial pseudogenes (numts; Song et al., 2008) was checked by translating the nucleotide sequences into protein sequences. The International Union of Pure and Applied Chemistry (IUPAC code) was used to determine heterozygosity (Alperi et al., 2008).

#### 2.1.4 Phylogenetic trees and networks

Phylogenetic trees of COI and 28S sequences were inferred using MrBayes 3.2.7 (Ronquist et al., 2012) through CIPRES Science Gateway v3.3 (Miller et al., 2010). For the best models of nucleotide substitution, GTR + G and TIM3 + I + G models were chosen for the 28S and COI dataset, respectively, based on the corrected Akaike information criterion (AICc) scores in jModeltest 2.1.10 (Darriba et al., 2012). However, the TIM3 model was not available for MrBayes; therefore, GTR, another overparameterized model, was used instead for the COI dataset (GTR + I + G). For all datasets, two independent runs, consisting of four chains each, were simultaneously conducted for 2,000,000 and 5,000,000 generations for 28S and COI sequences, respectively, with a sampling frequency of 1000. The initial 25% of samples were discarded. The resulting trees were visualized in FigTree v1.4.4. Also, phylogenetic networks of COI and 28S, as well as of ITS sequences, were produced based on HKY85 distances using a Neighbor‐Net method implemented in Splitstree5 (Bryant & Moulton, 2002; Huson & Bryant, 2006), to visually and effectively compare overall phylogenetic patterns. Sequences that were too short were excluded from the analyses because of these increased ambiguities which were represented by many boxes in the networks.

#### 2.1.5 Pairwise genetic distance

Uncorrected pairwise genetic distances of 28S and COI sequences were calculated in Mega7 using alignments used for phylogeographical patterns. Highly variable regions within 28S sequences were useful to distinguish groups but also included indels which made some parts of the alignment ambiguous. Therefore, we obtained pairwise genetic distances using two different datasets: one with only a highly conserved region (432 bp) and another with the full length of the amplicon (~1760 bp).

### 2.2 Species delimitation

We used three of the widely used molecular species delimitation methods for the COI and 28S datasets, respectively. The General Mixed Yule‐Coalescent model (GMYC) requires an ultrametric tree as an input and identifies a transition between speciation and coalescence processes by identifying a shift in the branching patterns (Pons et al., 2006). Ultrametric trees of 28S and COI sequences were generated in BEAST 2.6.6 through CIPRES Science Gateway (Miller et al., 2010) with a Yule model and a relaxed clock as an assumption. 10 million iterations of Markov Chain Monte Carlo (MCMC) were used and trees were sampled every 1000 MCMC iterations. Convergence and estimated effective sample sizes (ESS > 200) for all the parameters were checked using Tracer v1.7.1 (Rambaut et al., 2018). Trees were summarized using TreeAnnotator v2.6.6 with 10% burn‐in (Drummond & Rambaut, 2007). GMYC was conducted by using the splits package implemented in R (version 3.5.2). Another tree‐based method, Bayesian Poisson Tree Processes (bPTP) was implemented in the web interface (https://species.h‐its.org/ptp/) with 500,000 generations with 0.25 burnin (Zhang et al., 2013). ASAP (Assemble Species by Automatic Partitioning) is a method to build species partitions using pairwise genetic distances and was performed in the web interface with the default setting (https://bioinfo.mnhn.fr/abi/public/asap; Puillandre et al., 2021).

### 2.3 Substitution saturation

Substitution saturation was tested using Xia's method implemented in DAMBE7 (Xia et al., 2003). Xia's test compares the index of substitution saturation (*I*_ss_) and the critical index of substation saturation (*I*_ss.c_). *I*_ss.c_ is the point “at which the sequences will begin to fail to reconstruct the true tree” (Xia et al., 2003). If the *I*_ss_ value is not significantly smaller than the *I*_ss.c_ value, that suggests that sequences have experienced severe substitution saturation and there is little or no phylogenetic information. Because substitution saturation often occurs in the third codon position (Breinholt & Kawahara, 2013), the first and second codon positions, and the third codon position were tested separately. The tests were conducted for all the data, and each main lineage separately (N, C, SA, and SB). Separate analyses for each group allowed us to verify which group is likely responsible for the overall substitution saturation. In addition, substitution saturation was visually assessed by plotting transitions and transversions against K2P distance, also using DAMBE7.

### 2.4 Non‐random association between host mtDNA and intracellular parasites

#### 2.4.1 Sample collection, PCR, and sequencing

In order to see if host mtDNA and parasites are non‐randomly associated within a population, four populations were chosen. For selecting the populations, two criteria were considered: (1) have both Microsporidia and *Rickettsia* been found in the location? (2) do we have enough specimens collected from the location? Among the locations in the South Island meeting these two conditions, four populations were chosen to represent the major lineages (S15 and S40 for SA, S44 for SB, and S30). For each population, 2–4 appendages were obtained from 12–24 individuals to purify host genomic DNA. After obtaining appendages, the rest of the body was separately used for DNA extraction to be used for parasite detection. DNA was extracted using the Chelex method as described by Park et al. (2020) or with the Qiagen DNeasy Blood & Tissue kit following the manufacturer's protocol. Microsporidia and *Rickettsia* were detected as described in Park et al. (2020) and Park and Poulin (2020), respectively. The presence or absence of both parasites was recorded for each individual amphipod. After conducting PCR reactions for the COI region as described above, approximately the same number of infected and uninfected individuals were chosen for sequencing. All PCR products obtained in this study were purified with MEGAquick‐spin^TM^ Total Fragment DNA Purification Kit (iNtRON Biotechnology) according to the manufacturer's instructions. Purified PCR products were sent to Genetic Analysis Services at the University of Otago, New Zealand. Raw nucleotide sequences were carefully examined and corrected by eye, and the existence of nuclear mitochondrial pseudogenes (numts; Song et al., 2008) was checked by translating the nucleotide sequences into protein sequences.

#### 2.4.2 Phylogeny and haplotype networks

Another Bayesian COI tree was inferred to better observe non‐random association between endosymbiont infection status and mtDNA (i.e., COI haplotypes). For this, 64 sequences that we obtained from the four populations (S15, S30, S40, S44) were added to the dataset of the phylogeographical patterns. COI sequences from Hogg et al. (2006) and Lagrue et al. (2016) were also included, producing a final dataset of 128 sequences. Two independent runs, consisting of four chains each, were simultaneously conducted with GTR + G + I as a model of nucleotide substitution for 1,000,000 generations with a sampling frequency of 1000 in MrBayes. The initial 25% of samples were discarded. The resulting trees were visualized in FigTree v1.4.4. Additionally, median‐joining haplotype networks were drawn for each of the four populations (S15, S30, S40, and S44) using PopArt (Leigh & Bryant, 2015) to better observe associations between *Rickettsia* and host COI haplotypes. For the S40 population, Fisher's exact probability test was used to statistically test if one of the two genetic groups (S40_A and S40_B) within the same population (i.e., S40) has more infected individuals than the other (i.e., if the host genetic group and the infection status are non‐randomly associated).

## 3 RESULTS

### 3.1 Contrasting phylogeographical patterns

**FIGURE 1:**
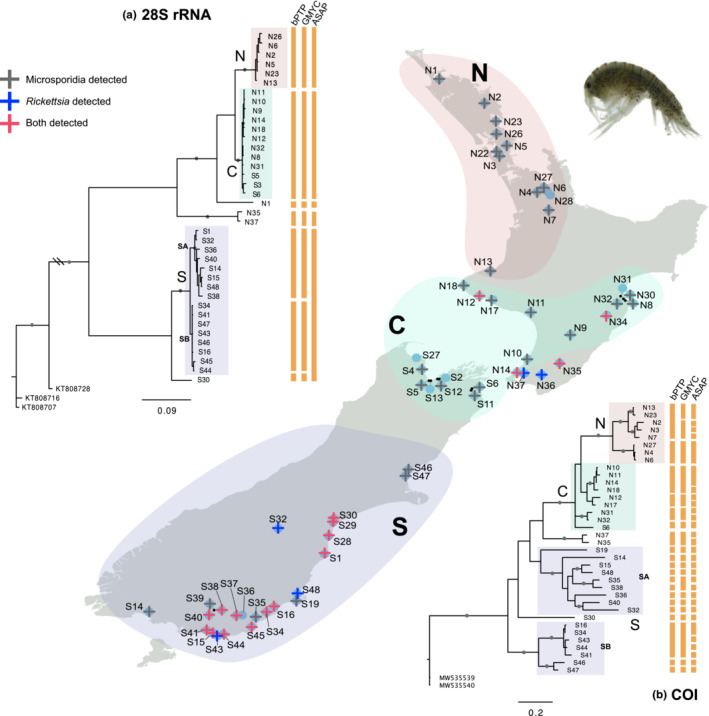
Map of New Zealand showing 59 sampling sites of *Paracalliope* with circles. The sites where parasites were detected are marked with crosses of different colors. Also, Bayesian trees of (a) 28S and (b) COI sequences are shown on the left and right sides of the map, respectively. Geographical regions are marked with different color shades on the map and the trees. Major lineages with high bootstrap support values (BS > 0.95) are highlighted with gray circles on the branch. The C and S groups are not monophyletic in the COI tree. The bars next to the trees show the results from the species delimitation methods (bPTP, GMYC, and ASAP).

**FIGURE 2:**
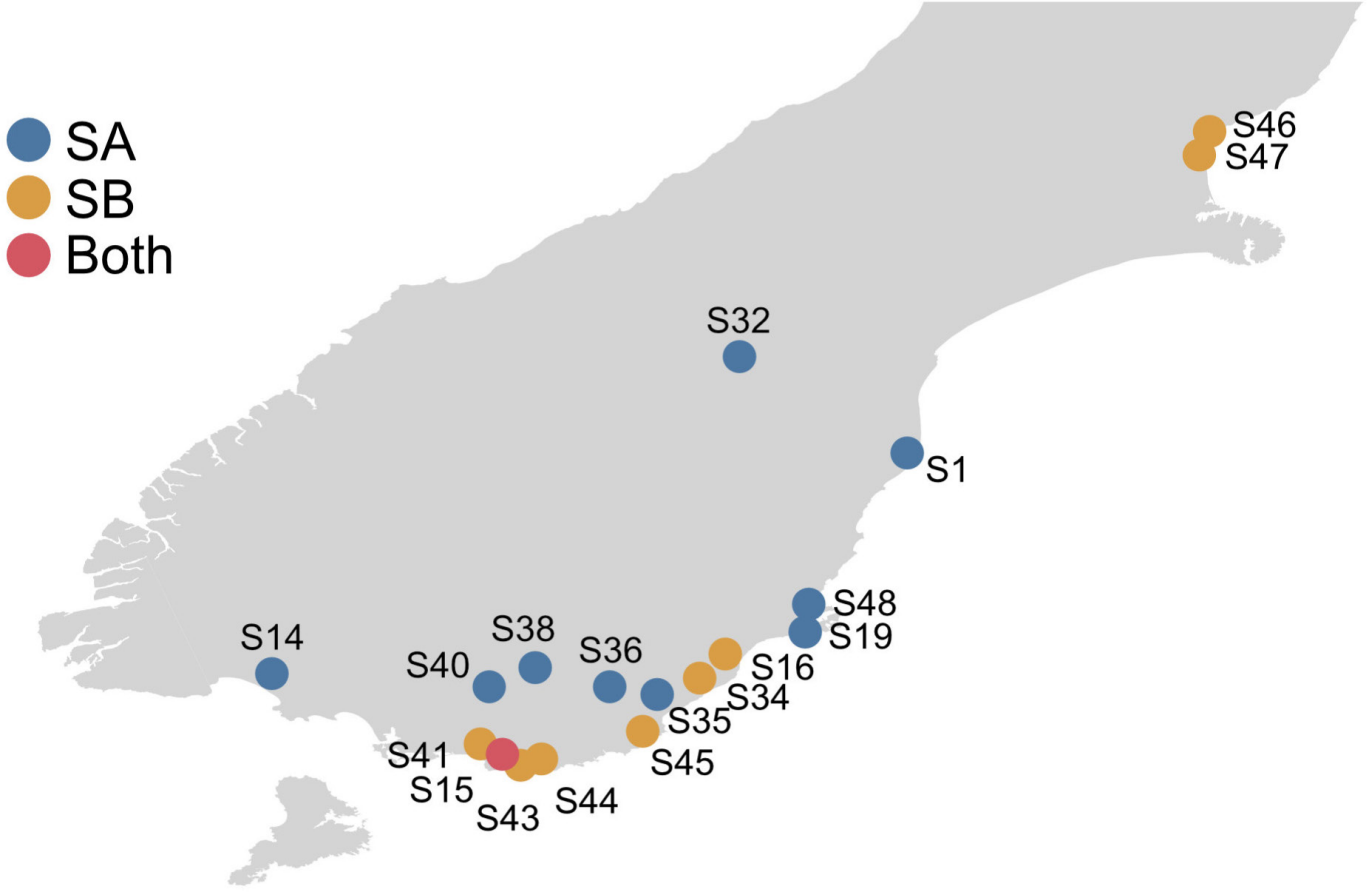
Map of the southern part of the South Island, New Zealand, showing the collection sites of SA and SB groups. Circles represent sampling locations. The SB group was found only in lower streams near the coastline, or a lake with saltwater intrusion. On the other hand, the SA group was found in upper streams as well as near the coastlines. Their distribution suggests that SA and SB groups may have originated from different marine ancestral lineages.

In total, 38 COI and 38 28S rRNA sequences (one sequence per one location) of *Paracalliope* were used to infer phylogenetic trees (Figure 1; see Table S1 for accession IDs and further information). Although both 28S and COI trees showed the same major groupings (N, C, SA, and SB), they showed different phylogeographic patterns. The phylogeographical patterns shown with 28S sequences well reflect known geographical events (see discussion). A clear split between the NC and S groups with large genetic distances suggests that these two main lineages diverged a long time ago from their most recent common ancestor. The 28S tree supports several independent freshwater invasions because freshwater lineages in the North and South Islands are not monophyletic but they are closely related to brackish lineages (e.g., N1, S30), respectively. The SB lineage has only been found near coastal areas in contrast to the SA group that was found in both inland and coastal areas (Figure 2).

**FIGURE 3:**
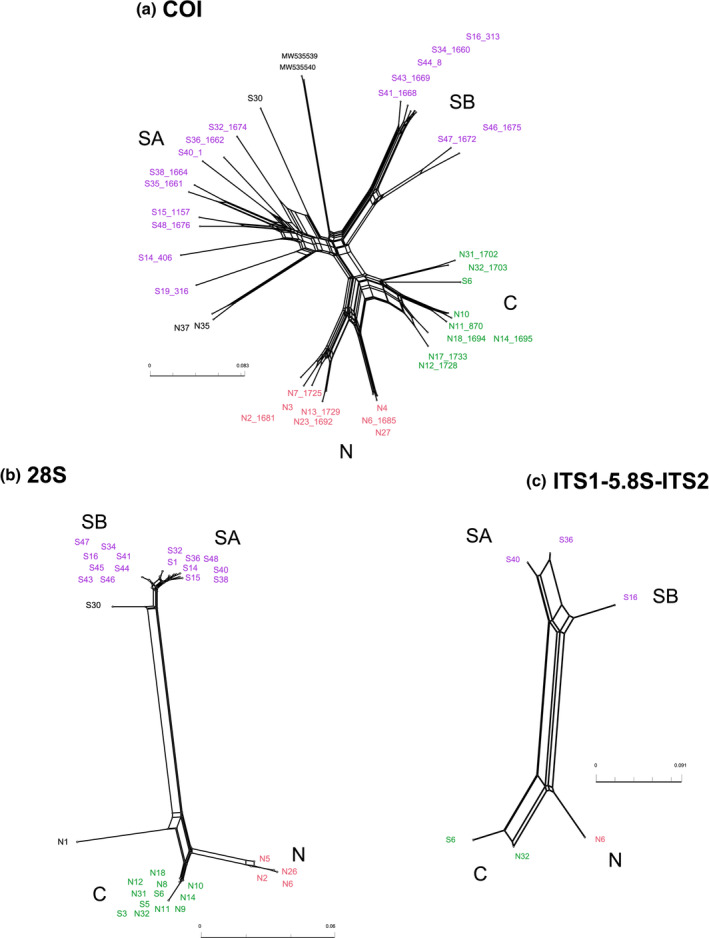
Phylogenetic networks of (a) COI, (b) 28S, and (c) ITS sequences. COI sequences are highly divergent especially in the SA group showing long terminal branches. 28S and ITS networks show a clear divide between the NC and S (SA and SB) groups.

In the COI tree, the C and S groups were not recovered as monophyletic (Figure 1). Also, branch lengths were considerably longer within the SA group compared to other groups. Some deep splits were also seen in the N, C, and SB groups, despite little variation in 28S sequences (Figure 1). We only obtained six clear sequences of ITS due to many repeats and indels. Although most of the ITS sequences we obtained were discarded because of low quality, the clear distinction among different major groups, which was similar to the pattern of 28S sequences, was observable. The different patterns between 28S and COI sequences are also clearly seen in the phylogenetic networks (Figure 3). The COI network shows extremely divergent lineages especially within the SA group (Figure 3a). In contrast, the 28S and ITS networks show clear distinction (long distance) between NC and S groups and little differentiation within each group (Figure 3b,c). Different patterns between mtDNA and nuDNA were also observed in the pairwise genetic distances of 28S and COI sequences (Tables [Link], [Link]). With the full length of 28S sequences which include highly variable sites, four main groups were distinguished (Table S2). The difference between the NC and S groups with the full 28S sequences was considerably high (15%–20%). While the genetic divergence within each group was very small (0%–1.2%), the genetic divergences between N and C groups were 4.5%–5.9%, and between SA and SB groups were 0.9%–2.8% (Table S2). Using only the conserved region of 28S sequences, the genetic distances between the NC and S groups were 6%–8% (Table S3). Unlike 28S sequences, which showed very little within‐group genetic distance, COI sequences show high intra‐ and intergroup genetic distances (Table S4). Notably, the SA group showed generally high genetic divergence (13%–24%) compared to that of other groups (2%–16%).

### 3.2 Species delimitation

Results from the three molecular species delimitation methods (bPTP, GMYC, ASAP) are shown next to the COI and 28S trees (Figure 1). Overall, the three methods show similar results for each gene. For 28S sequences, the two tree‐based methods (bPTP and GMYC) supported four species that correspond to N, C, SA, and SB groups. The ASAP method lumped SA and SB together supporting three freshwater species (Figure 1a). For COI sequences, all three methods supported higher number of putative species in freshwater lineages; 18, 17, 27, respectively (Figure 1b). Notably, bPTP and ASAP methods split all the sequences representing different locations in the SA group into different species (Figure 1b).

### 3.3 Substitution saturation

**FIGURE 4:**
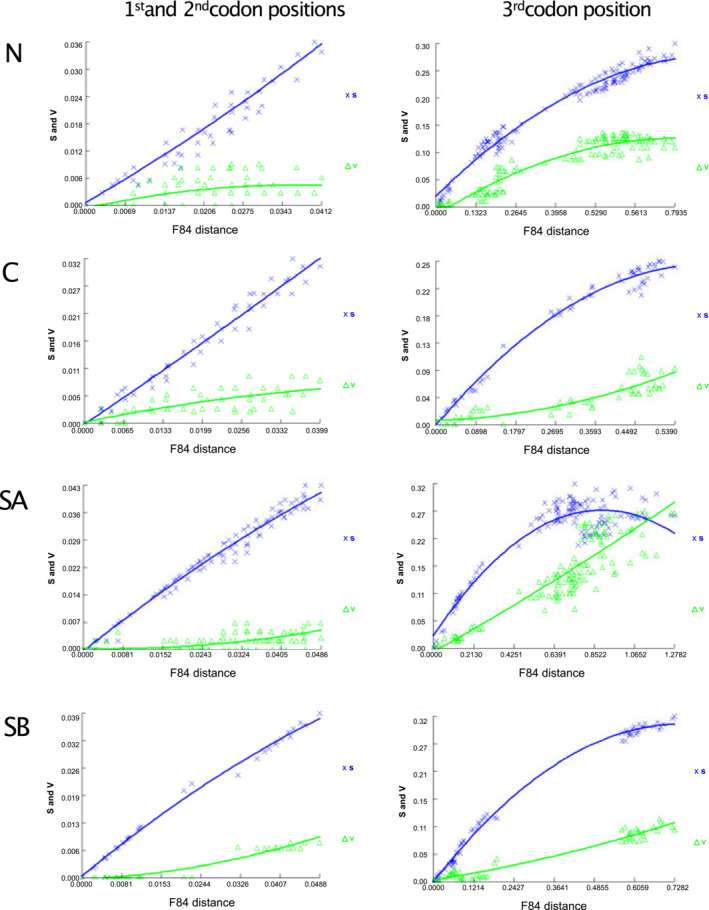
Plots of transitions and transversions against F84 genetic distance to visually diagnose substitution saturation. The plots were drawn for each group of *Paracalliope*, and for the first and the second codon positions, and the third codon position, separately. The third codon position of the SA group is saturated with transversions catching up transitions. S and V in the *y*‐axis represent transitions and transversions, respectively.

**FIGURE 5:**
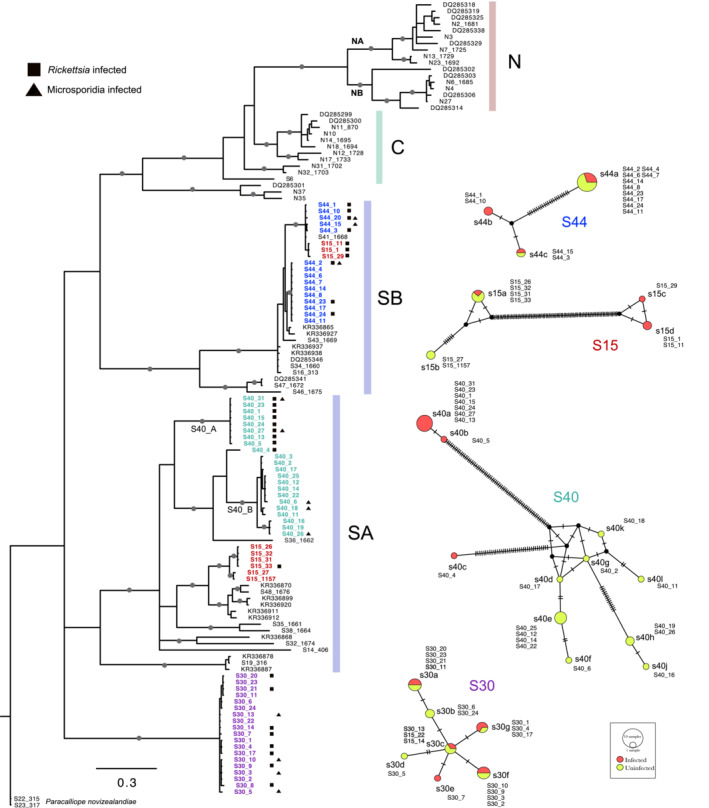
A Bayesian tree of COI sequences to show the linkage disequilibrium (LD) between parasites (Microsporidia and *Rickettsia*) and host COI haplotypes. The data on parasite infection status and host haplotype were obtained from four populations (S15, S40, S40, S44). Microsporidians do not show a clear pattern of LD, but *Rickettsia* shows clear LD in the S40 population. Haplotype networks are also shown for each population. Colors in the haplotype networks represent infection status (pink; infected, lime; uninfected).

Plots of transitions and transversions versus genetic distance estimated by the F84 substitution model show no saturation on the first and second codon positions in all the main lineages, with transitions higher than transversions (Figure 4). The third codon position of the N, C, and SB groups showed no saturation, however, in the SA group transversions caught up with transitions. This means that the same sites were likely to be affected by multiple hits, and therefore, this position has little phylogenetic information. The results of Xia's tests show that the third codon position is significantly saturated in New Zealand *Paracalliope* (*I*_ss_ > *I*_ss.c_, *p* < .0001); therefore, this position is useless for phylogenetic inference (Table 1). Although *I*_ss_ is not larger than *I*_ss.c_ in Xia's test for the third codon position of the SA group, they are not significantly different (*p* = .11), suggesting significant saturation (Xia et al., 2003). Xia's test suggests the third codon position of the N group is also saturated, but separate tests for the NA and NB groups show no saturation (see Figure 5 for NA and NB). Xia's tests also show that the first and the second positions are not saturated in any of the groups.

**TABLE 1:** Xia's test of substitution saturation shown separately for the first and second codon positions, and for the third codon position, in the main lineages of the amphipod *Paracalliope*.

	1st and 2nd			3rd		
	*I*_ss_	*I*_ss.c_	Probability	*I*_ss.c_	Probability
N	0.2979	0.7016	0	0.5999	0.6367	.6047
NA	0.2872	0.7655	0	0.4147	0.7365	0
NB	0.2683	0.7238	0	0.4472	0.7033	0
C	0.1532	0.7181	0	0.4004	0.6924	0
SA	0.125	0.7016	0	0.5721	0.6367	.1137
SB	0.1258	0.7096	0	0.3121	0.6717	0
All	0.247	0.695	0	0.786	0.686	.0393

Abbreviations: *I*_ss_, index of substitution saturation; *I*_ss.c_, critical *I*_ss_ value.

### 3.4 Association between parasite infection status and COI haplotype

In total, 64 COI sequences (=64 individual amphipods) were obtained from four populations (S15, S30, S40, and S44). The infection status by each parasite (Microsporidia and *Rickettsia*) in each individual is shown in Figure 5. There was no noticeable association between Microsporidia and COI haplotypes. On the other hand, a clear association between *Rickettsia* and COI haplotypes was shown in S40 (Figure 5). The non‐random association between the host COI haplotype groups (S40_A and S40_B) and infection status (infected or uninfected) was also supported by Fisher's exact test; *p* = 4.19e−06. Among nine infected individuals sequenced, seven individuals were associated with the same haplotype (s40a), and one with another haplotype (s40b), but only with one SNP different from s40a. One infected individual (S40_4) was associated with a haplotype distinct from all other haplotypes (s40c). All the uninfected individuals were associated with other variant haplotypes (s40d ~ l). Because both the SA and SB lineages were found in S15 (Figure 5), we additionally obtained 28S rRNA sequences. As a result, COI lineages correspond to the 28S lineages (i.e., both were either SA or SB), which means that the two lineages coexist in the same location.

## 4 DISCUSSION

Although mtDNA has been widely used for phylogenetic and phylogeographic studies, mtDNA can be influenced by direct and indirect selection that undermines its use as a neutral marker (Ballard & Whitlock, 2004). In most animals, mtDNA is transmitted only through females, and therefore, it reflects the evolutionary history of females (Hurst & Jiggins, 2005). Feminization caused by endosymbionts (e.g., Microsporidia) is well known in amphipods (Terry et al., 1998). Therefore, mtDNA patterns in amphipods are expected to be influenced by those endosymbionts. However, this factor (i.e., endosymbionts) that can have profound effects on their hosts' mtDNA has rarely (or never) been considered in phylogenetic and phylogeographic studies on amphipods. The COI marker, a short fragment of mtDNA, is the most commonly used marker for DNA barcoding and species delimitation. COI sequences of *Paracalliope* suggested the presence of 17–27 cryptic species, in contrast to 28S sequences which only supported 3–4 species in our study. Since two groups of endosymbionts (microsporidians and *Rickettsia*) were only recently reported but found to be widespread among *Paracalliope* populations (Figure 1), we aimed to observe a potential association between host mtDNA and the endosymbionts. Although we could not observe a general pattern, one of the four populations tested showed a clear non‐random association between host genetic groups and the infection status (S40 in Figure 5). Below, we discuss a potential role for endosymbionts affecting mtDNA patterns in amphipods and consider some alternative hypotheses (i.e., ancient hybridization and life‐history traits). Also, we discuss contrasting phylogeographic patterns and species hypotheses shown by 28S and COI sequences, and their implications.

### 4.1 Potential role for endosymbionts

Endosymbionts can disrupt population and phylogeographical patterns in mtDNA in various ways (Engelstädter & Hurst, 2009; Hurst & Jiggins, 2005; Jiggins, 2003). Several authors have suggested a potential role for *Wolbachia* and other symbionts in promoting speciation (Brucker & Bordenstein, 2012; Shoemaker et al., 1999; Werren, 1998). Here, we propose that endosymbionts may have played some roles in promoting genetic differentiation within and among populations. We suggest two possible mechanisms for this. First, reproductive manipulation caused by endosymbionts may affect gene flow among individuals and lead to reproductive isolation. It is well known in *Wolbachia*‐host associations that cytoplasmic incompatibility caused by endosymbionts can generate genetic barriers between infected and uninfected individuals, or between males and females infected with different strains (Bourtzis et al., 1996). Second, recurrent selective sweeps may accelerate substitution rates in a population, although this hypothesis has not yet been theoretically and empirically tested. For a neutral site, the possibility for fixation of a mutation is equal to the mutation rate (Kimura, 1977). However, positive selection can quickly fix a mutation in a population (Fitzpatrick et al., 2010). Selective sweep can rapidly increase the frequency of an allele in a population (Kim & Neilsen, 2004). The same applies to the cases of reproductive manipulators and associated mitochondrial haplotypes (Hurst & Jiggins, 2005). For example, a rare haplotype was rapidly fixed in *Drosophila* in the USA (Turelli et al., 1992). This has resulted in the fixation of a rare haplotype in a very short period, which would have taken a long time without a selective sweep. If another selective sweep occurs in the same population with another rare haplotype, and if this process is repeated, recurrent selective sweeps can make two populations diverge rapidly. Another complicating factor is that *Rickettsia* is not alone. There could be other endosymbionts that are not known yet, and their complex interactions within a population could generate various patterns. Different combinations of endosymbionts exert different selection pressures on different populations, which can result in a highly heterogeneous selective landscape and divergence among populations. *Rickettsia* of the Torix group are common in diverse invertebrates and frequent horizontal transmission has been inferred (Park & Poulin, 2020; Pilgrim et al., 2021). Therefore, recurrent selective sweeps by different strains are highly likely.

Alternatively, high genetic variation within and among populations might be simply explained by several amphipod life‐history traits that lower the effective population size. Effective population size (*N*_e_) is one of the most important parameters underpinning population dynamics and the effectiveness of selection relative to drift (Charlesworth, 2009; Nei & Tajima, 1981). A population will be more susceptible to genetic drift when the effective population size is small. First, fluctuating sex ratio throughout the year due to environmental sex determination (ESD) may lower the effective population size. In amphipods, ESD is believed to play an important role in allowing males hatched earlier than females to grow larger, which is advantageous for mating success (Bachtrog et al., 2014) (Guler et al., 2012; McCabe & Dunn, 1997; Nomura, 2002). Therefore, sex ratio periodically changes in an amphipod population with ESD. The effective population size can be estimated as *N*_e_ = 4*N*_m_ *N*_f_/(*N*_m_ + *N*_f_), where *N*_m_ is the number of males and *N*_f_ is the number of females (Nomura, 2002). There are many ways to estimate *N*_e_ (Ryman et al., 2019), though it is always lower when the sex ratio is not 1:1. Second, variation in fecundity also lowers the effective population size (Vucetich et al., 1997). Fecundity of female amphipods varies considerably among individuals and throughout the year (Bella et al., 1996; Cunha et al., 2000; Park & Poulin, 2021).

Endosymbionts and taxon‐specific life‐history traits are not mutually exclusive as causes of mitonuclear discordance, but may also act synergistically to accelerate substitution rates and result in substitution saturation. For example, endosymbionts can influence host fecundity and change host sex ratio (Dunn et al., 2001). In addition, the selective sweep caused by endosymbionts often reduces genetic diversity and lowers the effective population size within an infected population (Hurst & Jiggins, 2005; Johnstone & Hurst, 1996). Therefore, after a selective sweep, the population will be more likely to be affected by genetic drift, which can lead to the fixation of a rare mutation.

### 4.2 Alternative explanations: geographical isolation and hybridization

Although geographical barriers and events may have played important roles in shaping the current distribution of divergent genetic lineages in amphipods (Adams et al., 2018; Hogg et al., 2006), the presence of divergent lineages within a single location cannot be explained by geographical barriers. Also, geographical isolation should have affected both mtDNA and nuDNA by reducing gene flow among populations unless secondary contact has occurred (Hogner et al., 2012; Zhang et al., 2019). The possibility that ancient hybridization events caused by secondary contact have homogenized nuDNA but not mtDNA cannot be ruled out (Fuertes Aguilar et al., 1999). Although freshwater amphipods have limited dispersal abilities, historical connections among different catchments or recent river capture due to changes in drainage geometry (Burridge et al., 2006; Carrea et al., 2013; Waters et al., 2020), or occasional flooding may have allowed gene flow among *Paracalliope* populations. If individuals from one population migrate into another population, they may coexist in the same habitat. This may explain the presence of divergent COI lineages in sympatry. In addition to this “ancient hybridization” scenario, below we focus on some potential factors that may have affected mtDNA and nuDNA differently, namely “vertically transmitted endosymbionts” and “amphipod‐intrinsic factors.”

### 4.3 Implications: deeply divergent COI sequences do not necessarily suggest the presence of “cryptic species”?

Deep genetic divergence is often interpreted as a result of prolonged isolation (hence low or no gene flow between populations is inferred), and/or it provides a basis for species delimitation (Hebert et al., 2003; Ratnasingham & Hebert, 2013). When deeply diverged mtDNA lineages are not supported by nuclear markers and cannot be distinguished morphologically, mitonuclear discordance is associated with “cryptic species” (Bickford et al., 2007).

The presence of divergent COI lineages in New Zealand freshwater *Paracalliope* has been shown in several studies and the presence of cryptic species has been suggested earlier (Hogg et al., 2006; Lagrue et al., 2016; Sutherland et al., 2009). Despite several attempts made by amphipod taxonomists, meaningful morphological differences have not been detected among *Paracalliope* individuals from different habitats within New Zealand (Hogg et al., 2006; Sutherland et al., 2009) and among different continents (Chilton, 1920). *Paracalliope* has been discovered in freshwaters in India, the Philippines, New Caledonia, and Tasmania (Chilton, 1920; Iannilli & Ruffo, 2007; Knott, 1975), suggesting their ancient origin possibly dating back to Gondwana. Chilton (1920) reported that he could not find any morphological differences between amphipod specimens of *Paracalliope* from New Zealand, India, and the Philippines. If this “genus” indeed has an ancient origin, this would be a striking example of morphological stasis similar to only a few cases shown in some arthropods and annelids (Cerca et al., 2020; Szudarek‐Trepto et al., 2021). Even within New Zealand, the genetic divergence between NC and S groups was considerably high (15%–20% based on the 28S sequences; Table S2) suggesting morphological stasis of this taxon. The estimated substitution rates for 28S are in the range of 0.13%–0.3% Ma^−1^ (Copilaş‐Ciocianu et al., 2019). If we apply these rates, the estimated divergence time between NC and S lineages would be 66–153 MYA. This means that even though some amphipod individuals are indistinguishable based on their morphology, their genetic divergence can be very high, even exceeding that at species, genus, or family level in other taxa. Notably, the divergence level between NC and S groups based on COI sequences was ~24%, which is not very different from that of 28S sequences. Therefore, COI sequences “underestimate” the level of divergence between NC and S lineages. The 3rd codon position of the COI sequences was saturated even within a single group (SA), suggesting that this position does not contain phylogenetically informative information. The high intra‐ and intergroup divergence (hence long internal and terminal branches) of COI sequences explain the poor performance of the species delimitation methods which identify changes in branching patterns (tree‐based methods) or barcoding gaps (distance‐based methods). Therefore, the COI gene has little use for phylogenetic studies for this species complex.

In *Paracalliope*, it seems that phylogeographic patterns shown by 28S sequences well reflect the known geographical histories (e.g., marine transgression, recolonization of the newly available land area, and the last glacial maximum that have shaped the geographical distributions of many other taxa in New Zealand [Park et al., 2020]). In addition, 28S trees better explain a mating experiment conducted by Sutherland et al. (2009) in which the authors tested whether mate discrimination proportionally increases with genetic distances (COI) in *Paracalliope*, using 7 divergent populations from both the North and South Islands. Despite high COI divergence (19.5%) between two populations (Hamilton and Napier; belonging to N and C groups, respectively), individuals from these populations paired and produced eggs. However, individuals from North and South populations (divergence >21.5%) tended not to pair when in the presence of each other; the few individuals that did pair did not produce eggs, suggesting behavioral and genetic isolation between North and South Island populations.

Although deep genetic divergence is often interpreted as evidence for the existence of cryptic species (Bickford et al., 2007; Fišer et al., 2018), there are numerous examples where highly divergent mtDNA suggests the presence of cryptic species, while nuDNA, behavioral, and morphological data do not support cryptic species (Giska et al., 2015; Hinojosa et al., 2019; Pazhenkova & Lukhtanov, 2019). In amphipods, divergent COI lineages were not supported by morphology among Australian chiltoniid amphipods (King et al., 2012). Mitonuclear discordance between COI and ITS sequences was observed in *Niphargus* in Austria (Stoch et al., 2020). In Iceland, five distinct COI clades were identified in the groundwater amphipod *Crangonyx* (Kornobis et al., 2010), but ITS sequences and nuclear genomic data (ddRAD) showed different patterns and did not support cryptic species (Eme et al., 2017; Kornobis & Pálsson, 2011).

### 4.4 Caveats and future directions

We observed interesting contrasting patterns between COI and 28S sequences, and a non‐random association between the host COI group and *Rickettsia* in one population that suggest a potential impact of endosymbionts on their host mtDNA. However, several clear limitations exist in our study, and therefore, further investigation of certain unresolved issues would be valuable. Firstly, providing direct evidence of endosymbionts impacting their host reproduction (i.e., feminization caused by endosymbionts) and sex ratio change in *Paracalliope* would be crucial to support our endosymbiont hypothesis. Secondly, contrasting phylogeographic patterns with the use of multiple nuclear and mitochondrial genes would more clearly show mitonuclear discordance patterns. Furthermore, genomic data can be used to infer demographic histories which are needed to disentangle various causes that can also explain mitonuclear discordance patterns. Also, obtaining both mtDNA and nuDNA from multiple individuals per location would more clearly show the diversity patterns. Thirdly, the incidence and diversity of endosymbionts in other amphipods need to be quantified to see if endosymbionts have indeed played some roles in host mtDNA evolution. Also, understanding how different strains/species/groups of endosymbionts interact at a population level would inform on further conclusions regarding their evolutionary consequences. In conclusion, mtDNA has been a source of useful information to resolve the evolutionary history of organisms. However, mtDNA and nuDNA have inherently different natures and are likely to show different phylogenetic patterns. Also, because mtDNA is maternally transmitted, it is prone to be affected by co‐transmitted endosymbionts. Because of widespread morphological stasis and homoplasy and the presence of diverse endosymbionts, using multiple markers will be especially important in amphipods, and any mtDNA pattern should be interpreted with caution.

## AUTHOR CONTRIBUTIONS

**Eunji Park:** Conceptualization (equal); data curation (lead); formal analysis (lead); investigation (equal); visualization (lead); writing – original draft (lead); writing – review and editing (equal). **Robert Poulin:** Conceptualization (equal); investigation (equal); resources (supporting); supervision (lead); writing – review and editing (equal).

## CONFLICT OF INTEREST

The authors declare no conflicting interests.

## Supporting information


Table S1

Table S2

Table S3

Table S4

## Data Availability

All the sequences generated in this study are deposited in GenBank (Accession ID: MW535438–MW535501, MW535503–MW535540, MW537712–MW535717, MW537737–MW537748).
