# Supplementary material for: Extremely divergent COI sequences within an amphipod species complex: A possible role for endosymbionts?
Source: Ecol Evol. 2022 Oct 27;12(10):e9448. doi: 10.1002/ece3.9448 (PMC9609454; doi:10.1002/ece3.9448)
Supplement: Supplementary file 2 — Table S2 [file ECE3-12-e9448-s001.pdf]

Table 2. Uncorrected pairwise distance of 28S sequences of Paracalliope populations. A full length of amplicon was used.

	N	N	N	N	C	C	N	N	N	N	N	N	N	N	N	N		SA	SA	SB	SB	SB	SB	SB	SB	SB	SB	SA	SA	SA	SA	SA	SA	
	**N26**	**N6**	**N2**	**N5**	**S3**	**S5**	**N31**	**N32**	**N8**	**N11**	**N10**	**N9**	**N18**	**N14**	**N12**	**S6**	**N1**	**S1**	**S32**	**S34**	**S41**	**S47**	**S43**	**S46**	**S16**	**S45**	**S44**	**S36**	**S40**	**S14**	**S15**	**S48**	**S38**	**S30**
**N26**																																		
**N6**	0.4																																	
**N2**	0.2	0.2																																
**N5**	0.6	0.5	0.2																															
**S3**	5.4	5.9	5.1	4.7																														
**S5**	5.4	5.6	5.9	5.4	0.1																													
**N31**	5.1	5.7	5.1	4.5	0.7	0.1																												
**N32**	5	5.7	5.1	4.5	0.8	0.1	0.2																											
**N8**	5.7	6	5.2	5.2	0.4	0.1	0.1	0																										
**N11**	5	5.6	4.9	4.5	1	0.4	0.4	0.3	0.3																									
**N10**	5	5.4	4.9	4.6	0.3	0.5	0.3	0.3	0.3	0																								
**N9**	5.2	5.6	4.9	4.5	1.2	0.4	0.6	0.5	0.3	0.2	0																							
**N18**	4.9	5.6	4.9	4.4	0.8	0.4	0.4	0.4	0.4	0.2	0	0.4																						
**N14**	5	5.6	4.9	4.5	1	0.5	0.4	0.3	0.3	0	0	0.2	0.2																					
**N12**	5.2	5.9	5.1	4.5	0.9	0.3	0.4	0.5	0.4	0.3	0.1	0.5	0.3	0.3																				
**S6**	5.4	6	5.1	4.8	0.7	0.1	0.5	0.5	0.3	0.7	0.3	1	0.7	0.7	0.7																			
**N1**	10	10.1	7.7	9.7	7	7.6	7	7	7	7.3	7.3	7.3	7.3	7.3	7.2	7																		
**S1**	22.7	21.4	15.7	20.3	20.9	18.6	20	20	18.7	20.1	16	21.1	20.2	20	21	20.2	16.6																	
**S32**	18	17.9	15.4	16.6	15.6	17.6	15.6	15.6	16	15.8	15.8	15.8	15.8	15.8	15.6	15.6	17	0.3																
**S34**	22	20.6	14.9	19.8	20.2	18.1	19.7	19.6	17.9	19.7	15.5	20.4	19.7	19.6	20.2	19.8	16.7	1.5	0.8															
**S41**	17.6	17.6	15.1	16.5	15.6	17.3	15.6	15.6	15.8	15.7	15.6	15.7	15.7	15.6	15.6	15.6	16.7	1.1	0.8	0														
**S47**	22	20.6	14.9	19.8	20.2	18.1	19.6	19.6	17.9	19.7	15.5	20.4	19.7	19.5	20.2	19.8	16.7	1.5	0.8	0.1	0													
**S43**	22	20.6	14.9	19.8	20.2	18.1	19.7	19.6	17.9	19.7	15.5	20.4	19.7	19.6	20.2	19.8	16.7	1.5	0.8	0.1	0	0.1												
**S46**	21.9	20.6	14.9	19.7	20.1	18.1	19.7	19.5	17.9	19.6	15.5	20.3	19.6	19.5	20.2	19.7	16.7	1.4	0.8	0.1	0	0.1	0.1											
**S16**	17.7	17.7	15.1	16.4	15.4	17.3	19.7	15.4	15.7	15.6	15.6	15.6	15.6	15.6	15.4	15.4	17.4	1.2	0.9	0	0	0	0	0										
**S45**	21.3	20.2	14.8	20.1	19.5	17.6	19.7	19.5	17.6	19.7	15.1	19.7	19.7	19.5	19.6	19.8	16.2	2.2	0.9	0.4	0.4	0.4	0.4	0.4	0.2									
**S44**	21.5	20.5	15.9	21.1	20.4	17.9	19.7	20.5	18.3	20.7	16.2	20.7	20.7	20.5	20.6	20.7	16.5	2.3	0.8	0.2	0.1	0.2	0.2	0.2	0	0.2								
**S36**	22.4	20.9	15.5	20.3	20.9	18.3	19.7	20.2	18.5	20.3	15.8	21.1	20.4	20.2	21	20.4	16.6	1	0.9	1.8	1.5	1.7	1.8	1.6	1.5	1.9	2.2							
**S40**	23	21.7	15.7	20.4	21.2	18.8	19.7	20.1	19	20.2	16.1	21.4	20.3	20.1	21.3	20.3	17.1	1.1	1.2	1.6	1.7	1.5	1.6	1.5	1.7	2.2	2.5	0.9						
**S14**	22.3	20.9	15.4	20.1	20.9	18.5	19.7	19.9	18.3	20	15.9	20.8	20.1	19.9	20.6	20.2	16.8	1.5	1.4	1.9	2.1	1.9	1.9	1.8	2	2.4	2.8	1.2	1					
**S15**	22.4	20.9	15.5	20.1	20.9	18.5	19.7	19.8	18.5	20	16.1	21	20.1	19.9	20.9	20.1	17	1.2	1.4	1.7	1.8	1.6	1.7	1.6	1.8	2.1	2.4	1	0.8	0.8				
**S48**	22.4	20.9	15.6	20	20.9	18.6	19.7	19.8	18.5	19.9	16	21	20	19.8	20.8	20.1	16.9	1.2	1.4	1.7	1.8	1.6	1.7	1.6	1.8	2.1	2.4	0.9	0.8	0.6	0.2			
**S38**	22.1	21.3	15.4	20.8	20.4	18.6	19.7	20.5	18.7	20.7	16	20.7	20.7	20.5	20.6	20.6	16.9	1.6	1.1	2.1	1.5	2.1	2.1	2.1	1.5	2.1	2.4	0.9	1.1	1.2	0.7	0.6		
**S30**	21.6	20.9	14.4	19.6	19.8	18.5	19.7	19.2	18	19.2	15.7	19.8	19.4	19	19.6	19.4	16.2	6.2	3.1	5.7	3.5	5.6	5.7	5.6	2.5	5.2	5.4	6.2	6.1	6.3	6.2	6.3	6.4	
