# Supplementary material for: Extremely divergent COI sequences within an amphipod species complex: A possible role for endosymbionts?
Source: Ecol Evol. 2022 Oct 27;12(10):e9448. doi: 10.1002/ece3.9448 (PMC9609454; doi:10.1002/ece3.9448)
Supplement: Supplementary file 3 — Table S3 [file ECE3-12-e9448-s003.pdf]

Table 3. Uncorrected pairwise genetic distance of 28S sequences of *Paracalliope* populations. Only a conserved region was used.

	N	N	N	N	N	C	C	C	C	C	C	C	C	C	C	C	C		SA	SA	SB	SB	SB	SB	SB	SB	SB	SB	SA	SA	SA	SA	SA	SA	
	**N26**	**N6**	**N2**	**N5**	**N23**	**S3**	**S5**	**N31**	**N32**	**N8**	**N11**	**N10**	**N9**	**N18**	**N14**	**N12**	**S6**	**N1**	**S1**	**S32**	**S34**	**S41**	**S47**	**S43**	**S46**	**S16**	**S45**	**S44**	**S36**	**S40**	**S14**	**S15**	**S48**	**S38**	**S30**
**N26**																																			
**N6**	0.0																																		
**N2**	0.0	0.0																																	
**N5**	0.3	0.3	0.2																																
**N23**	0.0	0.0	0.0	0.2																															
**S3**	1.4	1.2	1.2	1.4	1.2																														
**S5**	1.4	1.2	1.4	1.7	1.4	0.0																													
**N31**	1.4	1.2	1.2	1.4	1.2	0.0	0.0																												
**N32**	1.4	1.2	1.2	1.4	1.2	0.0	0.0	0.0																											
**N8**	1.4	1.2	1.2	1.4	1.2	0.0	0.0	0.0	0.0																										
**N11**	1.4	1.2	1.2	1.4	1.2	0.0	0.0	0.0	0.0	0.0																									
**N10**	1.4	1.2	1.2	1.4	1.2	0.0	0.0	0.0	0.0	0.0	0.0																								
**N9**	1.4	1.2	1.2	1.4	1.2	0.0	0.0	0.0	0.0	0.0	0.0	0.0																							
**N18**	1.4	1.2	1.2	1.4	1.2	0.0	0.0	0.0	0.0	0.0	0.0	0.0	0.0																						
**N14**	1.4	1.2	1.2	1.4	1.2	0.0	0.0	0.0	0.0	0.0	0.0	0.0	0.0	0.0																					
**N12**	1.4	1.2	1.2	1.4	1.2	0.0	0.0	0.0	0.0	0.0	0.0	0.0	0.0	0.0	0.0																				
**S6**	1.4	1.2	1.2	1.4	1.2	0.0	0.0	0.0	0.0	0.0	0.0	0.0	0.0	0.0	0.0	0.0																			
**N1**	2.2	1.5	2.1	2.3	2.1	2.3	2.6	2.3	2.3	2.3	2.3	2.3	2.3	2.3	2.3	2.3	2.3																		
**S1**	7.7	6.2	6.8	7.1	6.8	7.5	8.9	7.5	7.5	7.8	7.5	7.5	7.5	7.5	7.5	7.5	7.5	8.1																	
**S32**	7.4	5.8	6.6	6.8	6.6	7.3	8.6	7.3	7.3	7.5	7.3	7.3	7.3	7.3	7.3	7.3	7.3	7.8	0.2																
**S34**	7.4	6.2	6.6	6.8	6.6	7.3	8.6	7.3	7.3	7.5	7.3	7.3	7.3	7.3	7.3	7.3	7.3	8.3	0.7	0.5															
**S41**	7.4	6.2	6.7	6.9	6.7	7.4	8.6	7.4	7.4	7.5	7.4	7.4	7.4	7.4	7.4	7.4	7.4	8.3	0.7	0.5	0.0														
**S47**	7.4	6.2	6.6	6.8	6.6	7.3	8.6	7.3	7.3	7.5	7.3	7.3	7.3	7.3	7.3	7.3	7.3	8.3	0.7	0.5	0.0	0.0													
**S43**	7.4	6.2	6.6	6.8	6.6	7.3	8.6	7.3	7.3	7.5	7.3	7.3	7.3	7.3	7.3	7.3	7.3	8.3	0.7	0.5	0.0	0.0	0.0												
**S46**	7.4	6.2	6.6	6.8	6.6	7.3	8.6	7.3	7.3	7.5	7.3	7.3	7.3	7.3	7.3	7.3	7.3	8.3	0.7	0.5	0.0	0.0	0.0	0.0											
**S16**	7.4	6.2	6.6	6.9	6.6	7.3	8.6	7.3	7.3	7.5	7.3	7.3	7.3	7.3	7.3	7.3	7.3	8.3	0.7	0.5	0.0	0.0	0.0	0.0	0.0										
**S45**	7.4	6.2	6.6	6.8	6.6	7.3	8.6	7.3	7.3	7.5	7.3	7.3	7.3	7.3	7.3	7.3	7.3	8.3	0.7	0.5	0.0	0.0	0.0	0.0	0.0	0.0									
**S44**	7.4	6.2	7.2	7.5	7.2	8.0	8.6	8.0	8.0	8.0	8.0	8.0	8.0	8.0	8.0	8.0	8.0	8.3	0.8	0.5	0.0	0.0	0.0	0.0	0.0	0.0	0.0								
**S36**	7.1	5.8	6.4	6.7	6.4	7.1	8.4	7.1	7.1	7.3	7.1	7.1	7.1	7.1	7.1	7.1	7.1	8.1	0.5	0.2	0.2	0.2	0.2	0.2	0.2	0.2	0.2	0.3							
**S40**	7.1	5.8	6.4	6.6	6.3	7.1	8.4	7.1	7.1	7.3	7.1	7.1	7.1	7.1	7.1	7.1	7.1	8.1	0.5	0.2	0.2	0.2	0.2	0.2	0.2	0.2	0.2	0.3	0.0						
**S14**	7.1	5.8	6.4	6.6	6.3	7.1	8.4	7.1	7.1	7.3	7.1	7.1	7.1	7.1	7.1	7.1	7.1	8.1	0.9	0.7	0.7	0.7	0.7	0.7	0.7	0.7	0.7	0.8	0.5	0.5					
**S15**	7.4	5.8	6.6	6.9	6.6	7.3	8.6	7.3	7.3	7.5	7.3	7.3	7.3	7.3	7.3	7.3	7.3	8.3	0.7	0.5	0.5	0.5	0.5	0.5	0.5	0.5	0.5	0.5	0.2	0.2	0.2				
**S48**	7.4	5.8	6.6	6.8	6.6	7.3	8.6	7.3	7.3	7.5	7.3	7.3	7.3	7.3	7.3	7.3	7.3	8.3	0.7	0.5	0.5	0.5	0.5	0.5	0.5	0.5	0.5	0.5	0.2	0.2	0.2	0.0			
**S38**	7.1	5.8	6.4	6.6	6.3	7.1	8.4	7.1	7.1	7.3	7.1	7.1	7.1	7.1	7.1	7.1	7.1	8.1	0.5	0.2	0.2	0.2	0.2	0.2	0.2	0.2	0.2	0.3	0.0	0.0	0.5	0.2	0.2		
**S30**	6.1	5.9	5.3	5.5	5.2	6.0	7.3	6.0	6.0	6.2	6.0	6.0	6.0	6.0	6.0	6.0	6.0	6.1	3.1	2.9	2.6	2.7	2.6	2.6	2.6	2.6	2.6	2.9	2.9	2.9	2.4	2.6	2.6	2.9	
