# Supplementary material for: Extremely divergent COI sequences within an amphipod species complex: A possible role for endosymbionts?
Source: Ecol Evol. 2022 Oct 27;12(10):e9448. doi: 10.1002/ece3.9448 (PMC9609454; doi:10.1002/ece3.9448)
Supplement: Supplementary file 4 — Table S4 [file ECE3-12-e9448-s002.pdf]

Table 4. Uncorrected pairwise genetic distance of COI sequences among *Paracalliope* populations

	N	N	N	N	N	N	N	N	N	C	C	C	C	C	C	C	C			SA	SB	SB	SB	SB	SB	SB	SB	SA	SA	SA	SA	SA	SA	SA	SA	
	**N10**	**N14**	**N11**	**N18**	**N12**	**N17**	**N31**	**N32**	**S6**	**N13**	**N23**	**N2**	**N3**	**N7**	**N27**	**N4**	**N6**	**N37**	**N35**	**S19**	**S16**	**S34**	**S43**	**S44**	**S41**	**S46**	**S47**	**S14**	**S15**	**S48**	**S35**	**S38**	**S36**	**S40**	**S32**	**S30**
**N10**																																				
**N14**	0.4																																			
**N11**	1.7	1.5																																		
**N18**	3.5	2.8	3.9																																	
**N12**	9.5	9.7	9.7	9.5																																
**N17**	8.0	8.0	8.8	8.3	4.7																															
**N31**	12.6	12.1	13.0	12.9	12.7	12.3																														
**N32**	11.7	11.4	12.1	12.3	12.3	11.5	2.7																													
**S6**	12.0	12.8	12.4	12.6	13.2	12.3	13.3	12.1																												
**N13**	18.0	17.9	17.7	17.4	17.6	16.2	17.4	16.5	17.1																											
**N23**	18.5	18.6	18.5	17.8	18.0	16.3	18.0	17.4	18.0	0.9																										
**N2**	17.1	17.7	17.0	16.5	17.1	16.1	17.3	16.4	17.1	7.9	8.3																									
**N3**	17.6	17.9	17.6	17.1	17.7	17.3	16.7	16.5	18.0	6.8	7.4	4.7																								
**N7**	17.0	17.3	17.3	17.1	18.3	16.7	17.3	16.4	16.7	6.5	7.4	5.3	4.7																							
**N27**	16.6	15.7	16.6	17.0	14.4	14.7	16.6	15.8	17.6	12.8	13.1	13.8	14.4	12.9																						
**N4**	17.0	16.0	17.0	17.4	14.5	15.2	17.3	16.7	17.7	13.6	14.0	14.4	15.0	13.8	0.8																					
**N6**	17.3	16.0	17.3	17.7	15.2	15.5	17.7	17.0	18.3	13.5	13.8	14.8	15.5	13.9	0.9	0.8																				
**N37**	19.5	19.7	20.3	21.1	18.8	19.2	20.0	19.5	19.1	22.7	23.4	22.6	22.9	21.2	21.1	21.4	21.8																			
**N35**	20.3	20.5	21.2	21.5	19.8	18.9	20.5	20.0	19.1	22.4	23.2	22.6	22.6	21.2	21.3	21.5	22.0	4.8																		
**S19**	20.5	20.5	20.9	21.5	22.0	20.8	20.3	19.7	20.0	21.7	22.5	20.3	21.8	19.8	21.7	21.8	22.0	17.3	18.2																	
**S16**	21.1	20.9	21.8	21.4	22.0	20.8	22.1	22.1	20.2	21.7	22.3	22.4	22.6	22.9	23.9	23.8	23.9	23.5	23.2	23.3																
**S34**	20.8	20.5	21.5	21.1	21.8	20.6	22.0	22.0	20.3	21.5	22.3	22.6	22.7	23.0	24.0	23.9	24.1	23.6	23.3	23.5	0.5															
**S43**	21.2	21.0	21.7	21.1	21.7	20.5	22.1	21.8	20.8	22.0	23.2	23.3	23.5	23.3	23.4	23.3	23.5	23.8	23.3	24.4	3.3	2.9														
**S44**	20.6	20.7	21.1	20.5	21.6	19.8	21.6	21.8	20.8	21.9	22.3	22.7	22.6	22.9	23.3	23.2	23.4	22.9	22.7	23.8	2.2	1.9	3.0													
**S41**	21.4	21.0	21.4	21.1	21.2	20.9	22.0	21.7	21.4	23.2	24.1	23.8	23.8	24.7	23.7	23.6	23.8	24.4	24.2	24.1	5.0	4.5	4.7	4.2												
**S46**	20.9	21.2	21.7	21.4	20.8	20.2	20.9	20.5	21.1	23.0	24.0	22.0	22.9	23.0	22.6	22.9	23.3	21.7	21.4	23.5	15.3	15.5	16.8	16.0	16.7											
**S47**	20.6	20.5	21.1	20.8	20.3	19.7	20.6	19.7	20.5	22.6	22.9	22.6	23.6	23.3	23.1	23.3	23.8	22.0	22.6	22.9	15.0	14.8	15.6	15.4	15.3	6.7										
**S14**	22.0	21.6	22.7	22.3	23.3	22.6	21.8	20.9	22.3	21.1	21.2	21.5	21.5	20.3	23.1	23.3	23.5	19.1	19.7	18.0	23.0	23.2	22.9	22.9	23.6	22.7	22.0									
**S15**	20.8	20.5	21.2	21.2	21.2	20.8	20.8	20.5	22.6	22.4	23.6	21.1	21.8	21.4	21.9	22.4	22.6	18.0	18.6	17.1	20.6	20.8	21.4	21.3	20.9	21.7	20.8	16.4								
**S48**	20.7	20.7	21.0	21.0	21.8	21.0	21.5	21.1	22.4	21.0	21.7	19.4	20.2	19.7	22.0	22.6	22.8	18.4	18.2	18.2	21.1	21.5	22.4	21.9	21.1	21.6	21.3	15.1	7.8							
**S35**	20.6	20.4	20.0	20.8	23.3	21.4	21.4	21.6	21.4	22.6	23.7	23.7	23.5	23.0	22.9	23.5	23.7	20.6	20.0	19.8	22.2	22.2	21.8	22.0	21.4	24.1	23.7	16.3	13.2	13.6						
**S38**	21.4	21.0	20.9	21.5	21.8	20.9	20.9	21.1	22.6	20.8	22.3	21.5	21.1	21.5	22.8	23.0	23.2	21.4	21.4	19.7	22.7	22.4	22.3	22.7	22.1	23.8	23.5	17.6	14.8	14.3	6.4					
**S36**	20.3	19.9	20.8	20.3	21.5	21.7	21.7	20.6	21.7	22.4	22.7	21.4	21.7	21.7	21.1	21.8	22.1	18.8	17.9	18.9	22.6	22.7	22.6	22.4	21.7	22.4	23.2	18.0	13.9	15.0	17.1	17.3				
**S40**	21.9	22.2	22.8	22.0	22.5	21.9	21.7	21.1	24.1	23.2	23.4	22.2	23.0	23.8	23.1	23.5	23.6	18.4	19.2	17.6	23.6	23.6	23.2	22.6	23.6	22.2	22.7	18.7	14.7	16.4	16.0	15.5	14.2			
**S32**	22.3	21.8	22.7	22.0	21.5	21.4	20.3	20.2	21.7	23.3	24.5	21.5	22.6	22.1	22.2	22.3	22.4	19.5	20.6	17.9	23.0	23.0	22.6	22.7	22.9	22.4	22.1	18.5	17.3	16.4	17.1	17.7	17.7	14.7		
**S30**	21.7	22.0	21.9	21.7	21.9	21.7	21.1	21.7	21.1	22.8	23.4	23.0	23.8	23.1	23.2	22.8	23.1	19.5	20.7	21.2	23.4	23.4	23.3	22.7	22.8	24.1	23.0	22.2	20.1	20.4	22.2	21.2	21.9	20.8	17.9	
